# A novel approach to endophytic renal mass: Percutaneous indocyanine green injection for fluorescence-guided robot-assisted renal Tumorectomy

**DOI:** 10.1016/j.eucr.2026.103398

**Published:** 2026-03-04

**Authors:** Carlo Maria Scornajenghi, Pier Giorgio Nardis, Luca Matteo Gobbi, Ivan Di Giulio, Valerio Santarelli, Michele Di Dio, Bruno Bucca, Alessandro Sciarra, Francesco Del Giudice, Giovanni Di Lascio, Giorgio Franco, Giovanni Battista Di Pierro

**Affiliations:** a Department of Maternal Infant and Urologic Sciences, Sapienza University of Rome, Policlinico Umberto 1 Hospial, Rome, Italy; b Vascular and Interventional Radiology Unit, Department of Radiological Oncological and Anatomopathological Sciences – Policlinico Umberto I – Sapienza University of Rome, Rome, Italy; c Department of Urology, Annunziata Hospital - University of Calabria (Unical), Cosenza, Italy

**Keywords:** Indocyanine green, Fluorescence imaging, Partial nephrectomy, Renal tumor, Endophytic lesions

## Abstract

We report a novel fluorescence-guided approach for robot-assisted partial nephrectomy in a completely endophytic renal tumor. A 73-year-old woman with a 3-cm entirely endophytic left renal mass underwent ultrasound-guided percutaneous intratumoral indocyanine green injection after failure of selective arterial embolization. Near-infrared fluorescence imaging allowed accurate intraoperative localization and complete tumor enucleation. Operative time was 125 minutes, with a warm ischemia time of 25 minutes. The postoperative course was uneventful, renal function was preserved, and histopathology confirmed renal oncocytoma. Percutaneous intratumoral indocyanine green injection represents a safe and effective alternative for fluorescence-guided nephron-sparing surgery in anatomically challenging endophytic renal tumors.

## 1 Introduction

Endophytic renal masses represent a significant challenge in nephron-sparing surgery, as they are not readily visible on the renal surface. The evolution of surgical techniques, from laparoscopy to robot-assisted approaches, has undoubtedly enhanced the minimally invasive nature of these procedures. However, such advancements alone are insufficient to address the core difficulty of intraoperative tumor localization in cases of entirely endophytic lesions.^1^

To overcome this limitation, several adjunctive methods have been developed over the years, including intraoperative ultrasonography and the use of indocyanine green (ICG), either intravenously or via super-selective arterial embolization, to facilitate renal mapping through near-infrared fluorescence imaging with the Firefly® system.^2^

The Firefly® system is integrated into the da Vinci X® robotic platform and allows real-time visualization of near-infrared fluorescence, enabling intraoperative identification of indocyanine green signal through rapid switching between white light and fluorescence imaging modes of the endoscope.

At our institution, ICG-based embolization is routinely employed to improve intraoperative visualization of endophytic renal tumors. Nevertheless, this technique requires the technical feasibility of selectively accessing and embolizing the arterial branch supplying the tumor, which is not always achievable, and it also involves higher costs and technical complexity.^2^

We present the case of a 73-year-old female patient with a 3-cm completely endophytic renal mass located in the left kidney. In this patient, super-selective arterial embolization with ICG was technically unfeasible due to anatomical constraints. As an alternative, we employed a novel approach not previously reported in the literature: percutaneous intratumoral injection of ICG performed immediately prior to robot-assisted partial nephrectomy.

This innovative technique enabled precise intraoperative localization and complete excision of the lesion under near-infrared fluorescence guidance (NIRF) using the Firefly® imaging system.

## 2 Case presentation

**Fig. 1:**
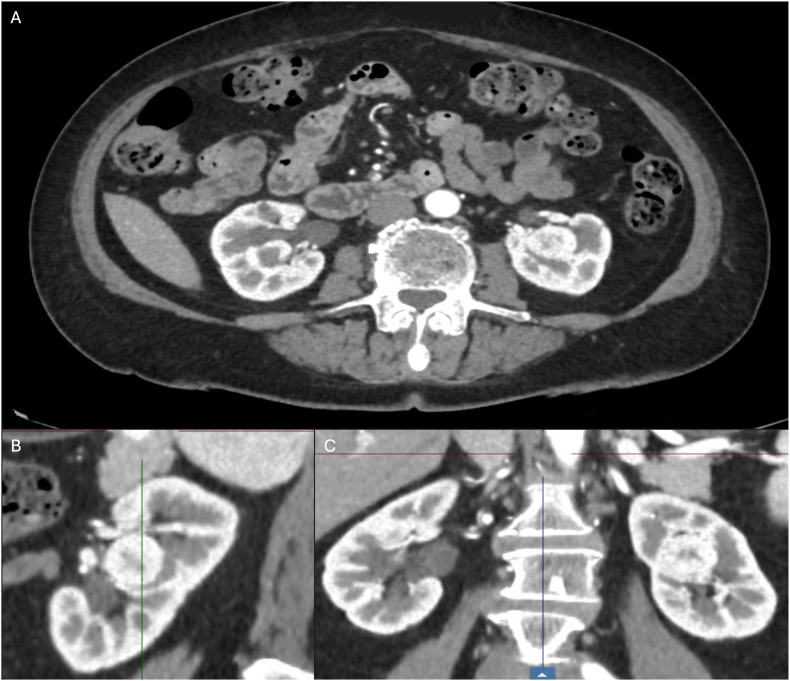
Arterial phase contrast-enhanced CT images of the left kidney showing a completely endophytic renal mass in axial (A), sagittal (B), and coronal (C) planes.

A 73-year-old female patient was referred to our department following the incidental detection of a renal mass during imaging performed for unrelated reasons. Contrast-enhanced CT revealed a 3-cm endophytic lesion located in the posterior aspect of the left kidney as shown in Fig. 1, classified as RENAL score 9p^3^ and PADUA score 10p.^4^ The mass showed no exophytic component and was entirely embedded within the renal parenchyma.

After multidisciplinary discussion, a decision was made to perform a robot-assisted partial nephrectomy (RAPN) with fluorescence guidance using ICG. An attempt at super-selective arterial embolization was made but failed due to the inability to catheterize feeder vessels.

Given the need for an alternative localization strategy, a percutaneous intratumoral injection of ICG was planned immediately prior to surgery.

The following day, under ultrasound guidance, a percutaneous injection of 1 ml of ICG (5 mg/mL) was performed directly into the lesion.

## 3 Technical description

### 3.1 Pre-operative phase

The procedure was performed under local anesthesia (2% mepivacaine). The patient was placed in the right lateral decubitus. In sterile settings under ultrasound guidance, a 22 G - 180 mm Chiba Type needle (HS-Italy) was advanced with the tip at the center of the nodule, through a single percutaneous puncture. Subsequently, 1mL of ICG 5mg/mL, powder in sterile water, was administered through the needle, resulting in a smooth hyperechoic change of the nodule while taking care to avoid ICG spillage onto healthy parenchyma. The needle was then removed and patient was transferred to the operating room.

In this specific case, the percutaneous injection was performed in a different procedural room for internal logistical reasons.

This type of percutaneous ultrasound-guided puncture is a well-known and safe procedure in urology, comparable to renal biopsy or fine needle-aspiration techniques.

### 3.2 Intra-operative phase

RAPN lasted 125 minutes without any deviation from the standard procedure. The patient was positioned in lateral decubitus. After standard trocar placement and medialization of the colon, the renal hilum was isolated, and the renal artery was identified. Complete mobilization of the kidney allowed full exposure of its posterior surface. The Firefly mode was initially unable to delineate the lesion clearly from the unincised posterior surface of the kidney. After clamping, a renotomy was performed on the posterior surface using a cognitive approach based on preoperative CT scan.

**Fig. 2:**
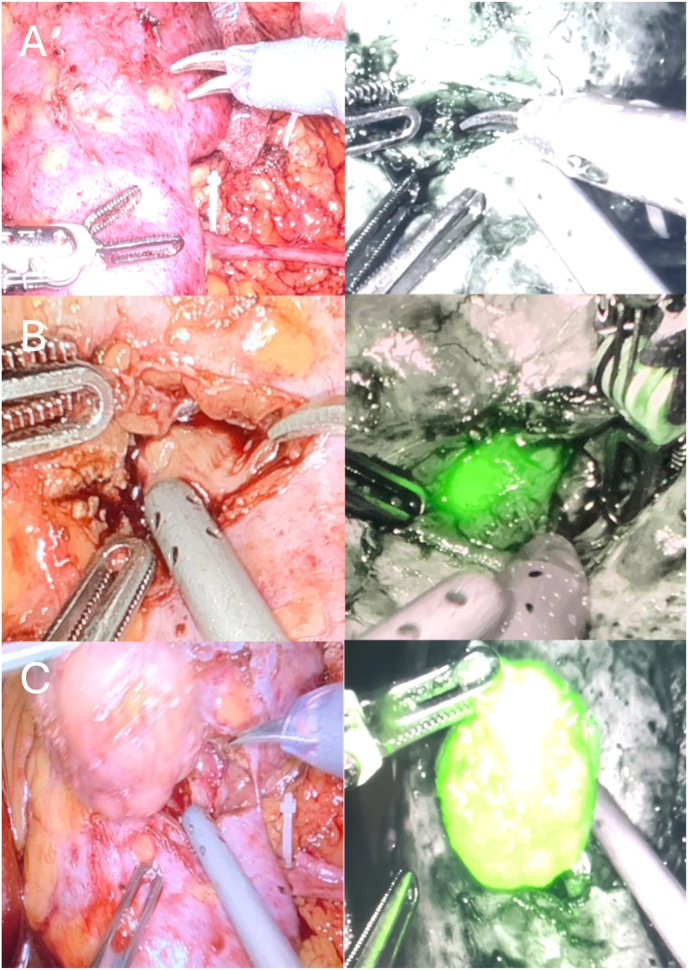
Intraoperative images during robot-assisted partial nephrectomy displayed in two columns, with white light imaging on the left and near-infrared fluorescence on the right. (A) The lesion is not visible under either modality on the intact renal surface. (B) After cognitive renotomy, the lesion remains undetectable under white light but is clearly identified using indocyanine green fluorescence. (C) The excised tumor is visible under both white light and near-infrared fluorescence.

The lesion became visible under near-infrared fluorescence using the Firefly system, allowing safe and complete enucleation (Fig. 2).

Hemostasis was achieved with monopolar coagulation and application of Oxytamp®. The cortical defect was closed with Monosyn® 2-0 sutures using sliding technique on Hem-o-lok® clips. Clamping time was 25 minutes, and a pararenal drain was placed.

### 3.3 Post-operative phase

Preoperative labs showed a baseline hemoglobin of 14.4 g/dL and serum creatinine 0.82 mg/dL. Postoperatively, hemoglobin remained stable at 13.7 g/dL on the first post operative day(POD I) setting a value of 13.0g/dL at discharge, and renal function was preserved (serum Creatinine 1.0 mg/dL), normalizing to 0.68 mg/dL by discharge. The patient had no significant complications.

The drain was removed on the POD II, after having drained totally 200mL sero-hematic fluid. The patient was discharged on POD III.

The histological examination confirmed a completely excised renal oncocytoma.

At the 3 months follow-up, an ultrasound evaluation was performed and showed no abnormalities with exception of the surgical scar.

## 4 Discussion

The pursuit of methodologies aimed at improving outcomes in the surgical management of renal masses, particularly those that are predominantly or entirely endophytic, has driven the development of various adjunctive technologies. Key tools include ultrasound-based techniques, transarterial embolization, dual-source computed tomography, invasive tumor localization methods, 3D printing, and augmented reality applications.

Intraoperative ultrasound (IOUS) remains a cornerstone technique in nephron-sparing surgery, offering high accuracy and oncologic safety in tumor localization. Its benefits have been demonstrated across multiple series, with low recurrence and acceptable positive margin rates, alongside improved outcomes as measured by the MIC (Margins, Ischemia, and Complications) composite score and Pentafecta achievement, when compared to the non-IOUS cohort. Multivariate logistic regression analysis further identified IOUS use during RAPN as an independent predictor for achieving MIC criteria.^5^^,^^6^

The use of ICG and its subsequent visualization through near-infrared fluorescence imaging in renal surgery has achieved significant success, largely due to its versatile applications. ICG can be introduced into the urinary tract via anterograde or retrograde approaches to comprehensively map its anatomy, or it can be employed within the vascular compartment. In the context of renal mass surgery, the latter application is of primary importance. Most commonly, ICG is administered intravenously during nephron-sparing surgery. The rationale for this approach is twofold: firstly, intravenous ICG preferentially stains the renal parenchyma over the tumor tissue, thereby delineating a clear margin between healthy and pathological tissue, which aids in minimizing collateral damage. Additionally, it facilitates a better understanding of renal vascularization, enabling selective clamping when necessary. Secondly, ICG can be used post-resection to assess the perfusion quality of the residual renal parenchyma.^7^ A more recent and highly promising application involves super-selective arterial embolization with ICG to selectively label renal tumors. This technique allows the use of near-infrared fluorescence imaging to localize even completely endophytic renal masses intraoperatively.^8^^,^^9^ Equally noteworthy are percutaneous techniques, which are particularly valuable in cases of renal neoplasms that are isoechoic relative to the renal parenchyma and thus difficult to identify using intraoperative ultrasound. Pietryga et al. (2012) demonstrated that the deployment of marker coils in tumors with poor visualization—such as intraparenchymal and isoechoic lesions—significantly facilitated computed tomography-guided percutaneous radiofrequency ablation (CT-PRFA). Specifically, their use resulted in a 58% reduction in CT fluoroscopy time, while maintaining technical outcomes comparable to those observed in patients without coil placement.^10^ Marker coils may also be visualized during intraoperative ultrasound, improving the localization of isoechoic masses; however, they offer limited advantage for non-isoechoic lesions, which constitute the majority. In this regard, the technique proposed by Ferakis et al., in 2022 is of particular interest. By adapting the use of hook-wire guidewires—commonly employed for marking pulmonary or breast tumors—they utilized a hook-wire to localize a cT1a endophytic renal mass prior to a three-dimensional laparoscopic partial nephrectomy (3D-LPN).^11^ The percutaneous use of ICG has been described primarily in the field of pulmonary neoplasms, where it has been successfully employed prior to thoracoscopic resection.^12^ This approach bypasses the challenges associated with selective angiography and embolization. The oncologic safety of percutaneous renal procedure performed with fine needles has been consistently confirmed in the literature. Needle tract seeding after renal mass biopsy is an extremely uncommon event, described almost exclusively in papillary renal cell carcinomas and in association with core needles of ≥20G or the absence of a coaxial sheath. Conversely, fine needles of 22G or smaller have shown a negligible risk of tumor cell dissemination, even in long-term follow-up studies.^13^ In our technique, a single percutaneous puncture was performed using a 22G Chiba needle exclusively for intratumoral injection of indocyanine green, without any aspiration or tissue retrieval.

The oncologic risk of tumor cell dissemination associated with this percutaneous technique appears comparable to or lower than that reported after percutaneous renal cryoablation, a setting in which needle tract seeding has been documented only in isolated case reports and small series.^14^

Motivated by these considerations, we adapted this technique into the realm of renal tumors to overcome the technical infeasibility of performing selective ICG embolization. To our knowledge, this represents the first case report describing the percutaneous injection of ICG into a renal mass.

The technique is inherently flexible and simple, allowing it to be performed in the operating room immediately prior to RAPN, permitting seamless integration into the surgical workflow without significant additional time.

Intraoperative visualization of the renal mass using the Firefly® system was optimal following a cognitive incision of the renal surface. Empirically, no leakage of ICG into the adjacent renal parenchyma was observed. RAPN was completed successfully without technical difficulties or complications attributable to percutaneous ICG administration. The postoperative course was uneventful, with no late complications related to either the percutaneous procedure or the robotic surgery.

This case demonstrates that percutaneous ICG injection is a feasible, safe, and effective alternative to arterial injection in select cases of endophytic renal tumors. It provided excellent intraoperative visualization and facilitated successful RAPN with optimal functional and oncologic outcomes. This method should be considered in similar anatomically challenging cases.

## CRediT authorship contribution statement

**Carlo Maria Scornajenghi:** Writing – original draft, Methodology, Investigation, Conceptualization. **Pier Giorgio Nardis:** Writing – review & editing, Resources, Methodology, Investigation. **Luca Matteo Gobbi:** Visualization, Investigation, Data curation. **Ivan Di Giulio:** Investigation, Data curation. **Valerio Santarelli:** Visualization, Investigation. **Michele Di Dio:** Writing – review & editing, Validation. **Bruno Bucca:** Investigation, Data curation. **Alessandro Sciarra:** Writing – review & editing, Supervision. **Francesco Del Giudice:** Writing – review & editing, Formal analysis. **Giovanni Di Lascio:** Investigation. **Giorgio Franco:** Writing – review & editing, Supervision, Project administration, Conceptualization. **Giovanni Battista Di Pierro:** Validation, Resources, Methodology.

## Ethical statement

The procedure was performed in accordance with institutional ethical standards and the Declaration of Helsinki. Written informed consent was obtained from the patient for both the procedure and publication.

## Funding

This research received no external funding.

## Conflict of interest

The authors declare no conflicts of interest related to this work.
